# Chasing Vibro-Polariton Fingerprints in Infrared and Raman Spectra Using Surface Lattice Resonances on Extended Metasurfaces

**DOI:** 10.1021/acs.jpcc.2c00779

**Published:** 2022-04-18

**Authors:** Francesco Verdelli, Jeff J. P. M. Schulpen, Andrea Baldi, Jaime Gómez Rivas

**Affiliations:** † Dutch Institute for Fundamental Energy Research, Eindhoven 5600HH, The Netherlands; ‡ Department of Applied Physics, Eindhoven University of Technology, Eindhoven 5600MB, The Netherlands; § Vrije Universiteit Amsterdam, Amsterdam 1081HV, The Netherlands; ∥ Institute for Photonic Integration, Department of Applied Physics, Eindhoven University of Technology, Eindhoven 5600MB, The Netherlands

## Abstract

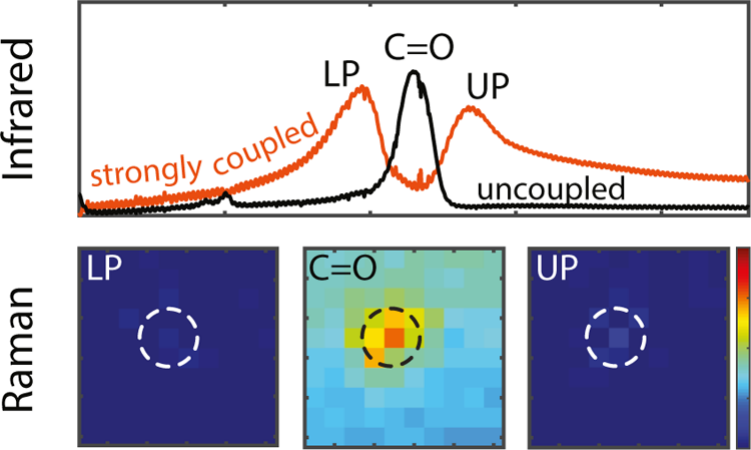

We present an experimental investigation of vibrational strong coupling of C=O bonds in poly(methyl methacrylate) to surface lattice resonances (SLRs) on arrays of gold particles in infrared and Raman spectra. SLRs are generated from the enhanced radiative coupling of localized resonances in single particles by diffraction in the array. Compared to previous studies in Fabry–Perot cavities, particle arrays provide a fully open system that easily couples with external radiation while having large field confinement close to the array. We control the coupling by tuning the period of the array, as evidenced by the splitting of the C=O vibration resonance in the lower and upper vibro-polaritons of the IR extinction spectra. Despite clear evidence of vibrational strong coupling in IR transmission spectra, both Raman spectroscopy and micro-Raman mapping do not show any polariton signatures. Our results suggest that the search for vibrational strong coupling in Raman spectra may need alternative cavity designs or a different experimental approach.

## Introduction

When light interacts with matter, it can generate a plethora of interesting phenomena with a variety of applications. This interaction is classified in terms of the energy exchange rate between light and matter given by the Rabi frequency, Ω_R_. If the optical losses or decoherence rates in the light-matter system are dominant over the energy exchange rate, the system is in a weak coupling regime. On the other hand, if this exchange rate is larger than the loss rate, the system is in a strong coupling regime.^1^ In this last regime, light and matter hybridize, leading to the formation of the so-called polaritonic states or polaritons, which are separated by the Rabi energy. These new hybrid states display properties of both photons and matter,^2^ distinct from the properties of the uncoupled systems. The strong coupling has opened new pathways for various applications.^3^ Notorious examples are sensing,^4^ lasing,^5−7^ exciton diffusion,^8−10^ charge transport,^11,12^ energy transfer,^13,14^ and catalysis.^15−17^

It has been shown that strong coupling between the electromagnetic modes in optical cavities with molecular vibrations or vibrational strong coupling (VSC) can also alter potential energy surfaces and modify reaction dynamics,^15,18^ both under illumination and in the dark. This intriguing possibility is receiving an increasing attention due to potential applications in chemistry^19−22^ and catalysis.^23,24^ Strong coupling to molecular vibrations has been demonstrated with modes in planar Fabry–Perot cavities,^25^ with propagating surface plasmon polaritons in metal films,^26^ 1D gratings,^27^ and with localized resonances in nanogap patch antennas.^28^ Recently, VSC has been also demonstrated in two-dimensional periodic arrays of resonant scatterers.^29^ These periodic systems sustain surface lattice resonances (SLRs). SLRs are the collective response of the scatterers in the array, mediated by the enhanced radiative coupling due to in-plane diffraction orders or Rayleigh anomalies (RAs).^30−33^ If the scatterers are metallic particles in the visible or near-infrared, the resonant response is provided by localized surface plasmons or the coherent oscillation of free charge carriers in the particles. These plasmonic metasurfaces provide high field enhancements with sub-wavelength mode volumes in fully open systems, and they are capable of achieving large light–matter coupling strengths,^34−36^ making them ideal platforms for the investigation of the effects of VSC on the Raman spectra of molecules. Shalabney *et al.* have recently shown that the intensity of the Raman signal is enhanced under VSC in a Fabry–Perot cavity, showing a Rabi splitting in the Raman spectrum.^37^ However, other recent studies do not observe the presence of the polariton bands in Raman spectra and only show a small signal enhancement of the molecular Raman peaks.^38^ These contradicting results motivate the investigation of VSC in open systems, such as metasurfaces or arrays of resonant scatterers, where Raman signals can be spatially resolved.

In this manuscript, we experimentally investigate the VSC between SLRs in gold metasurfaces formed by arrays of resonant gold microdisks and the C=O vibrational resonance in poly(methyl methacrylate) (PMMA) layers by measuring the IR extinction spectra. We have optimized the geometrical parameters of the metasurface with numerical simulations and spectrally overlapped the fundamental SLR to the C=O molecular vibration to achieve a maximum Rabi splitting of 80 cm^–1^, which is larger than the losses of the system.^39^ This Rabi splitting is further characterized by performing angular dispersion measurements and by detuning the SLR by changing the period of the array. We also investigate the Raman spectra of the coupled system using a confocal Raman microscope. Comparing the Raman spectra of bare PMMA and the strongly coupled system, we find no evidence of the signatures of vibro-polaritons in the Raman spectrum of the strongly coupled system. Finally, we map the Raman signals on the unit cell of the array at the energies of the upper and lower polaritons determined from the extinction measurements and of the C=O vibration to search for any local signature of VSC. However, while the far-field extinction measurements show clear upper and lower polariton bands, the Raman measurements do not show any evidence of the signatures of VSC.

## Sample Description

**Figure 1:**
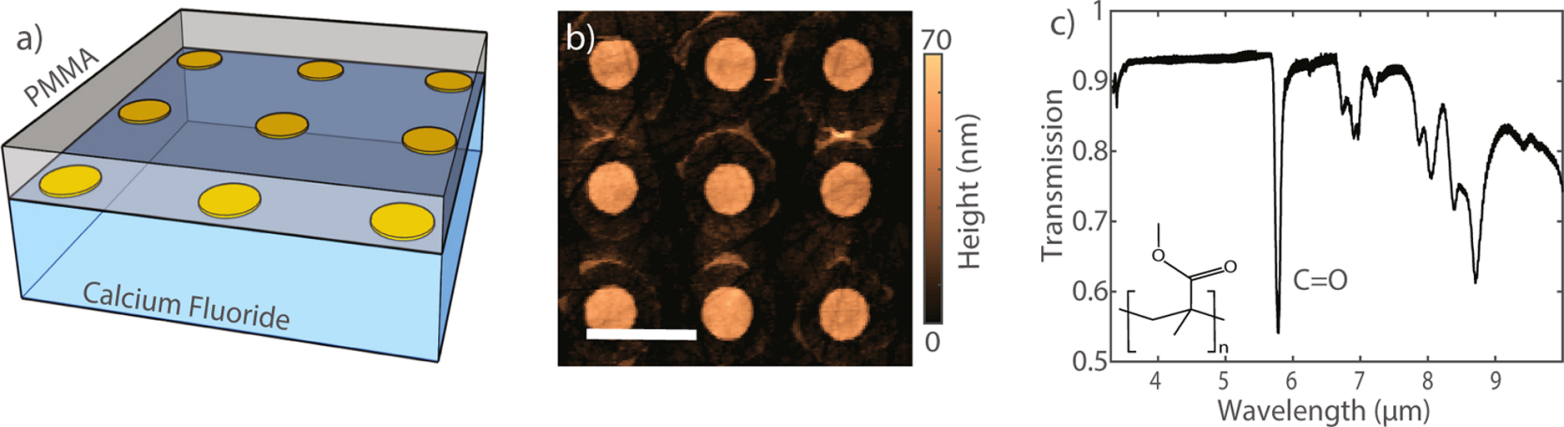
Gold microdisk arrays on a CaF_2_ substrate and PMMA characterization. (a) Schematic illustration of a sample consisting of a microdisk array on a calcium fluoride substrate and covered by a layer of PMMA. (b) Atomic force microscope image of a 3 × 3 particle region of one of the arrays. The scale bar indicates the length of the unit cell, which is 4 μm. The circle-like shadows around the disks are organic residues of the fabrication process that do not influence the array’s SLR. (c) FTIR transmission spectrum of a PMMA thin film. The C=O molecular resonance is indicated. The molecular structure of PMMA is shown as an inset.

Figure 1a shows a schematic representation of a metasurface that consists of a periodic square array of gold microdisks with a diameter of 1.4 μm on calcium fluoride (CaF_2_). The array is covered by a 1 μm thick PMMA layer. The microdisks are fabricated with optical lithography. A detailed description of this fabrication is given in the methods section. A top view of an array is displayed in Figure 1b using atomic force microscopy. This image reveals the precise shape, dimensions, and period of the array.

The arrays have been designed to have the fundamental SLR with an energy close to or resonant to the C=O bond of PMMA at normal incidence. Figure 1c displays a transmission measurement through a 1 μm thick layer of PMMA using Fourier transform infrared spectroscopy (FTIR). This measurement shows the C=O resonant absorption at 1732 cm^–1^.

## Methods

### Sample Fabrication

The following recipe was developed for a bright-field optical lithography mask. First, the calcium fluoride substrate was cleaned via immersion in an acetone bath for 5 min, followed by an isopropyl alcohol bath for 5 min, and rinsed with dH_2_O. After the cleaning step, the substrate was vapor-coated with HDMS to further improve the photoresist adhesion. The negative photoresist MaN-440 was spin-coated on the substrate for 30 s at 3000 rpm speed with an acceleration of 1000 rpm/s. It was then soft-baked on a hot plate for 120 s at 100°. The final photoresist film thickness was around 2 μm. The substrate was exposed using a Karl Suss MA-6 optical lithography at 365 nm for 100 s. This process was repeated 3 times with a 10 s pause in between exposures. The contact mode between the mask and the substrate was set to “vacuum”; this contact gives a resolution of the structures greater than 1 μm. The exposed sample was developed for 90 s in MaD-532s developer and then rinsed for 120 s in dH_2_O. An adhesion layer of 2 nm of Ti followed by a layer of 100 nm of Au was deposited using a BVR2008FC Electron Beam Evaporator. The evaporation rates for Ti and Au were 0.5 and 1 nm/s, respectively. The lift-off was performed in acetone. Finally, the sample was rinsed for 120 s in dH_2_O.

An 8% wt PMMA (Sigma-Aldrich) solution in anisole was spin-coated on the sample containing the microdisk arrays at 1000 rpm speed with an acceleration of 1000 rpm for 60 s. This process leads to a PMMA thin film thickness of 900 μm.

### Micro-Disk Array Characterization

The plasmonic array used in the measurements was tuned to have its SLR spectrally overlapping with the C=O molecular vibration of PMMA. This tuning was achieved through the optimization of three parameters: the array period, the disk diameter, and the thickness of the PMMA layer. In particular, the diameter of the single gold disk was optimized using finite-difference in time-domain (FDTD) simulations to align its LSPR to the RA of the array (Section S1). All these parameters were investigated with numerical simulations using FDTD simulations. In particular, we simulated a planar system consisting of a CaF_2_ substrate, the gold disks array with a 4 μm period, 1.4 μm disk diameter, and 50 nm disk height, and a 1 μm thin layer with the same real component of the refractive index as PMMA but without the molecular resonance. The simulated system was fabricated and measured in the FTIR with a GLOBAR light source using index matching oil with a refractive index of 1.44 and an upperstrate of CaF_2_. Each FTIR spectrum is the average of 20 single scans, taken 3 times per angle and averaged to reduce the noise level. Before determining the extinction, the measured transmission is normalized by the transmission through a sample without a microdisk array. The index matching oil does not present any molecular vibration in the spectral range of the C=O bond resonance.

### Raman measurements

The Raman spectra are measured with a Renishaw inVia microscope. For each array, the measurements are performed with an integration time of 5 min, and they are repeated three times for each array to improve the signal-to-noise ratio. The 2D maps of the Raman peak intensity are performed on an area of 5 μm × 5 μm with a 500 nm step size. These maps are performed on the array with a 4 μm period, with the same PMMA layer thickness used in the infrared measurements. For each step, the Raman spectrum is taken with an integration time of 5 min.

## Results and Discussion

### Characterization of the Bare MicroDisk Arrays

**Figure 2:**
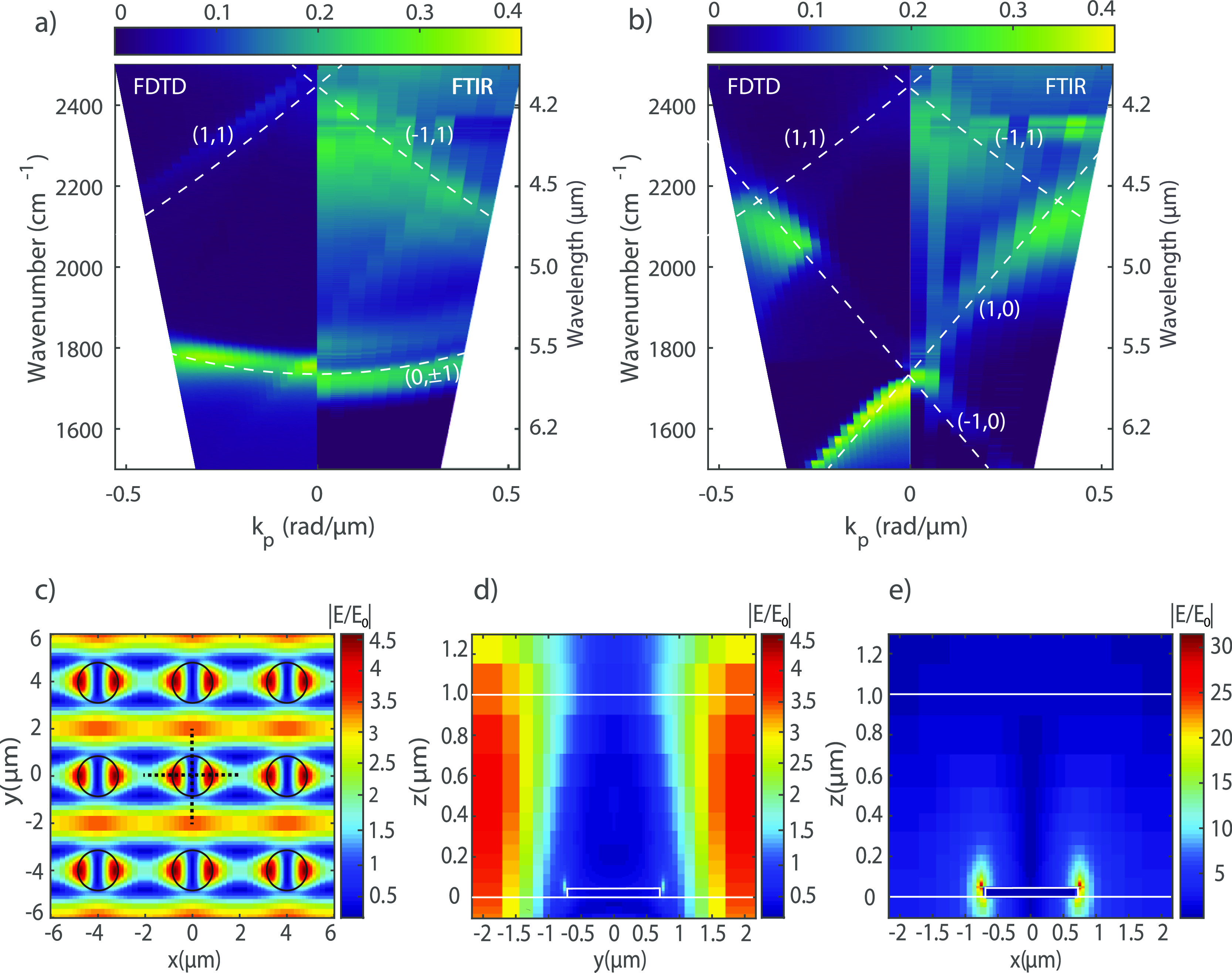
FDTD simulations and IR extinction measured with an FTIR spectrometer by varying the angle of incidence from normal up to 20° for (a) p-polarized light and (b) s-polarized light. (c) FDTD simulations of the amplitude enhancement of the scattered electric field *E*/*E*_0_ at the height of 400 nm above the substrate and wavenumber of 1732 cm^–1^ (*x*–*y* cross-section). The black dotted lines on the central particle show the position where the *y*–*z* cross-section and the *x*–*z* cross-section are investigated. Amplitude enhancement of the scattered electric field in (d) the *y*–*z* and (e) *x*–*z* planes at 1732 cm^–1^.

Before investigating the strongly coupled sample, we describe the bare metasurface without PMMA on top. We have simulated the extinction of the array using the FDTD method. These simulations are shown in the left panels of Figure 2a,b for different angles of incidence (θ) defining the wavenumber parallel to the surface of the array (*k*_p_ = (2π/λ) sin θ) and for the two polarizations p and s, respectively. For these simulations, we choose a period of the array of 4 μm. The disk height is set to 50 nm and the diameter to 1.4 μm. We consider a 1 μm thick layer of dielectric material with a refractive index *n* = 1.44 on top of the array and an upperstrate of CaF_2_ with the same dimension as the substrate. The dielectric material layer represents an index matching liquid that is used in the FTIR measurements to surround the microdisks with a homogeneous dielectric medium. This homogeneous surrounding facilitates the formation of SLRs since the in-plane diffraction orders responsible for the radiative coupling of the localized resonances in the individual microdisks are the same in the upper and lower media.

The corresponding extinction measurements, defined as 1 -transmission, are shown in the right panels of Figure 2a,b for p- and s-polarization, respectively. These measurements are performed in a Fourier transform infrared spectrometer by detecting the zeroth-order extinction at different angles of incidence of the infrared beam. Simulations and measurements of the bare metasurface show a similar dispersive extinction. The dashed white lines in panels 2a,b correspond to the in-plane diffraction orders or RAs of the array (see the Supporting Information Section S2). These RAs are calculated using the grating equation
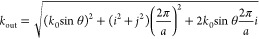
1where *k*_0_sin θ is the wavenumber projected on one axis, *a* is the period of the array, and *i* and *j* are the diffraction orders.

The bands of high extinction in Figure 2a,b are the SLRs for different polarizations, having the same dispersive behavior as the diffracted orders. The simulated and measured SLRs have slightly different frequencies, which could be explained by the nonperfect cylindrical shape of the microdisks (see the Supporting Information Section S3). Another possible cause for this shift is a small difference in the refractive index of the index-matching liquid in the IR range from its nominal value in the visible. In addition, the measured SLRs are slightly broader than the simulated ones. This difference is likely due to small fabrication imperfections and the limited spatial coherence of the IR source.^40^ The simulated data in Figure 2 are scaled in intensity by a factor of 0.4 to facilitate a comparison with the experimental data. The reduced extinction in the measurements is also a consequence of the imperfections in the array. Note that the diagonal feature visible in the measurements (right panel of Figure 2a) corresponds to the (1,0) diffraction order caused by a nonideal alignment of the IR polarizer.

The coupling strength of a coupled system depends on the electromagnetic field distribution of the SLR. Therefore the scattered electric field associated with the SLR has been simulated with FDTD using normal incidence and a beam polarized along the *x*-direction. These simulations are shown in Figure 2c–e. In Figure 2c, we display the SLR field in the *x*–*y* plane at a height *z* = 400 nm above the CaF_2_–PMMA interface. This field distribution is associated with the strong extinction due to the SLRs of the array.^41^ The fields on the *y*–*z* and *x*–*z* planes, displayed in Figure 2d,e, show that the SLRs are mostly confined to the PMMA layer, leading to an optimum situation for strong coupling.

### Characterization of the Coupled System

To achieve strong coupling, we spin-coated a PMMA layer with a thickness of 900 nm on top of the array. As for the bare array, the extinction is retrieved by measuring the transmission through the coupled system with FTIR for p- and s-polarizations. The measurements are then compared with simulations of the coupled system using FDTD.

**Figure 3:**
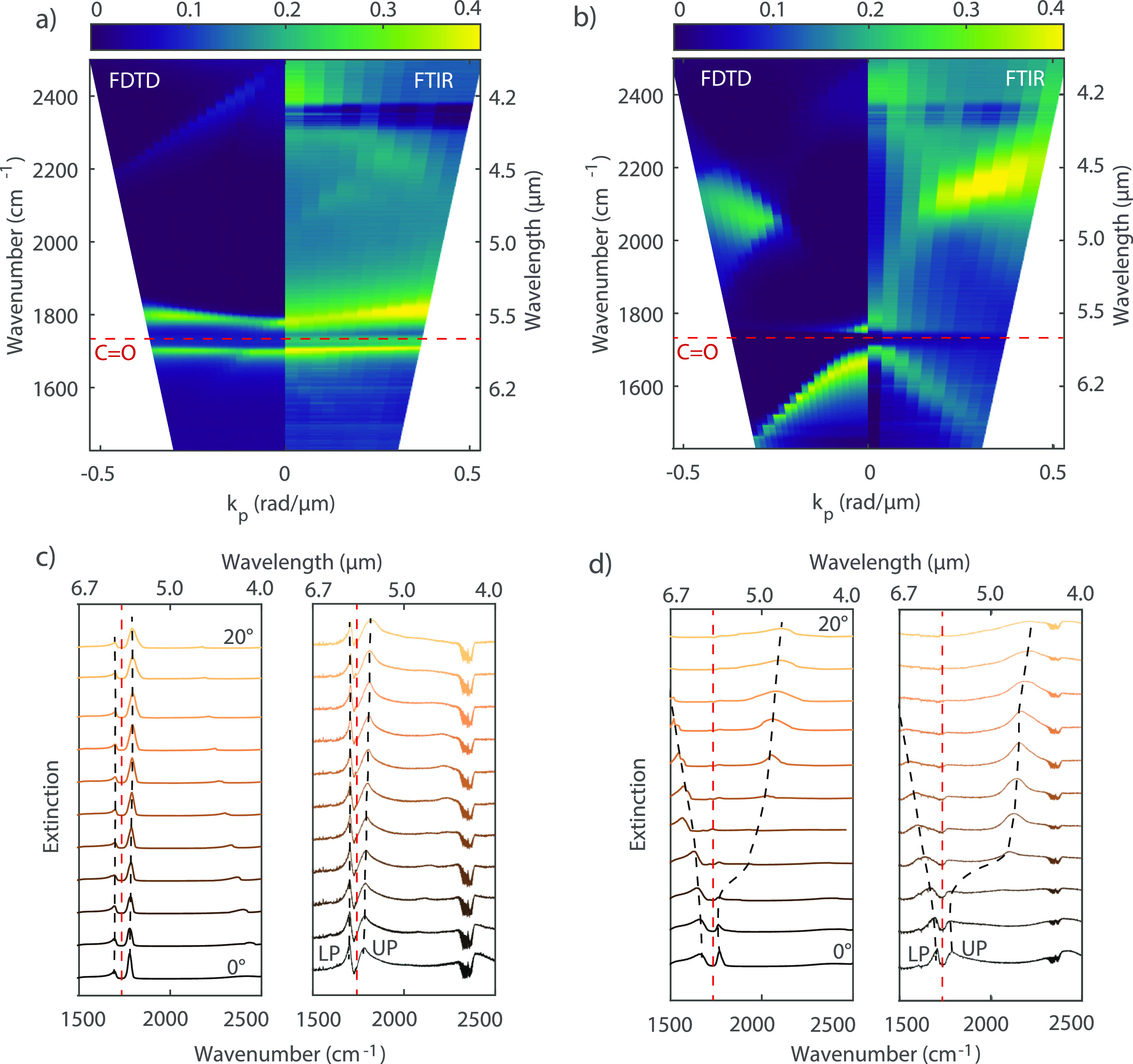
Extinction spectra of a strongly coupled array simulated and measured at different angles of incidence. The simulations are multiplied by 0.4 to facilitate the comparison. (a) Comparison between simulated and measured extinction for p-polarized light. (b) Comparison between simulated and measured extinction for s-polarized light. Polaritons are clearly visible for both polarizations around the C=O bond wavenumber (1732 cm^–1^) indicated by the red dashed line. (c) Simulated (left) and measured (right) spectra for different angles of incidence and p-polarized light. These spectra have been displaced vertically for clarity. The red dotted curve represents the spectral position of the C=O vibration. The black dotted lines are a guide to the eyes for the lower and upper polaritons. The feature in all spectra around 2400 cm^–1^ is the CO_2_ signature peak in the mIR. (d) Simulated (left) and measured (right) spectra for different angles of incidence and s-polarized light.

Figure 3a,b shows the simulated (left panels) and experimental (right panels) extinction dispersion for both polarizations, where a clear splitting in the extinction at the C=O bond energy and the concomitant lower and upper polariton bands around 1694 and 1770 cm^–1^ at *k* = 0 are observed. These two new states are generated by the coupling of the C=O vibration to the degenerate (0, ±1) SLRs (Figure 3a) or the (1,0) and (−1,0) SLRs (Figure 3b). Higher-order (1,1) and (−1,1) SLRs do not couple to the molecular vibrations, but they are still visible both for p- and s-polarizations. Figure 3c,d shows the simulated (left panels) and measured (right panels) extinction spectra for different angles of incidence. The two vibro-polaritons are visible above and below the C=O frequency (red dotted line) for both polarizations. The black dotted lines are guides to the eye, indicating the wavenumber of the lower and upper polaritons.

To confirm that the system is under the strong coupling regime, we retrieve the linewidth of the SLR (γ_SLR_ = 60 cm^–1^) from the FTIR measurements. The quality factor of the measured SLR is *Q* = 29. The PMMA C=O bond resonance width was measured to be γ_PMMA_ = 33 cm^–1^. The VSC condition is given by Ω_R_^2^ > (*γ*_SLR_^2^ + γ_C=O_^2^)/2.^1,39^ Therefore, the minimum Rabi energy splitting needed to achieve strong coupling is Ω_R_ = 46 cm^–1^. At normal incidence, we measured a Rabi splitting of Ω_R_ = 76 cm^–1^. This Rabi splitting shows that our array–PMMA system is in the strong coupling regime, and it can be used as a platform to investigate the Raman signal of the vibro-polariton bands.

**Figure 4:**
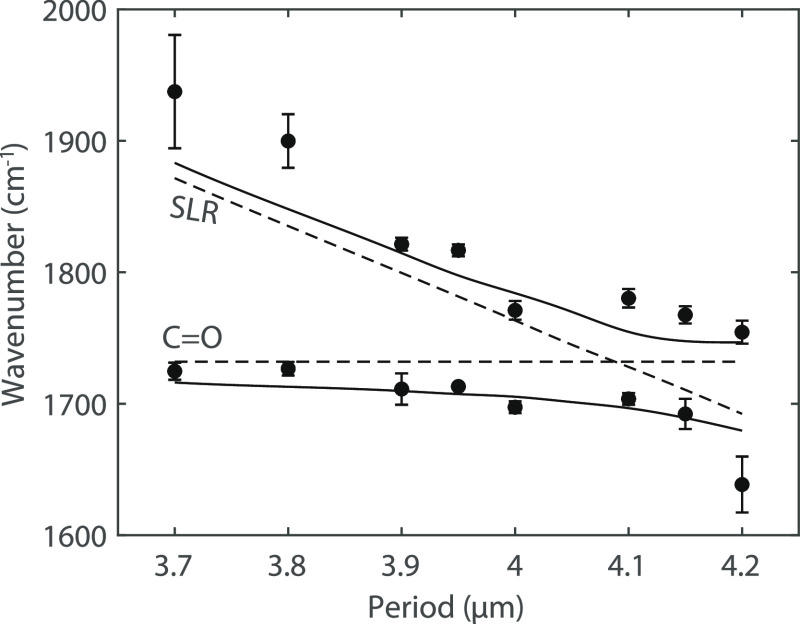
Polariton wavenumbers (solid circles) as a function of the period of the array defining the SLR detuning from the C=O bond. The dashed lines correspond to the C=O bond and the SLR energy. The solid curves represent the simulated upper and lower polariton energies.

It is also possible to recover the anticrossing behavior characteristic of strongly coupled systems by changing the period of the array and, therefore, the detuning between the SLRs and the molecular vibrations. For this purpose, we have measured the extinction at a normal incidence of a set of arrays with a period ranging from 3.7 to 4.2 μm (see the Supporting Information, Section S4). The frequencies of the upper and lower polaritons obtained from the extinction measurements are plotted in Figure 4 as a function of the period of the array (solid circles). The solid curves in the same figure represent FDTD simulations of the upper and lower polariton wavenumbers, and the dashed lines are the C=O and SLR wavenumbers. To determine the error in the measurements of Figure 4, two factors have been considered: the homogeneity of the arrays and the spectral resolution of the FTIR spectrometer. While the latter is a constant contribution to the error in each measurement, the former depends on fabrication imperfections of the arrays and thickness variations of the PMMA layer. We quantify this contribution to the error by measuring the arrays at different positions. A minimum splitting of 74 cm^–1^ is found for a period of 4.1 μm, which is similar to the value found at normal incidence with angular dispersion measurements.

### Micro-Raman Spectroscopy in VSC Samples

The signature of VSC in the Raman spectra has been recently reported in molecules strongly coupled to Fabry–Perot cavity modes.^37,42^ However, other studies have not found clear signatures of strong coupling in the Raman signal despite showing strong coupling in the IR spectra.^38^ In contrast, theoretical works suggest that the Rabi splitting of a strongly coupled system should be present in the Raman spectra, with the lower and upper polariton located at the same energies and with the same linewidth as in the IR spectra.^42,43^ Motivated by these contradicting results, we have investigated the presence of polaritons in the Raman spectra of the strongly coupled molecules to SLRs.

**Figure 5:**
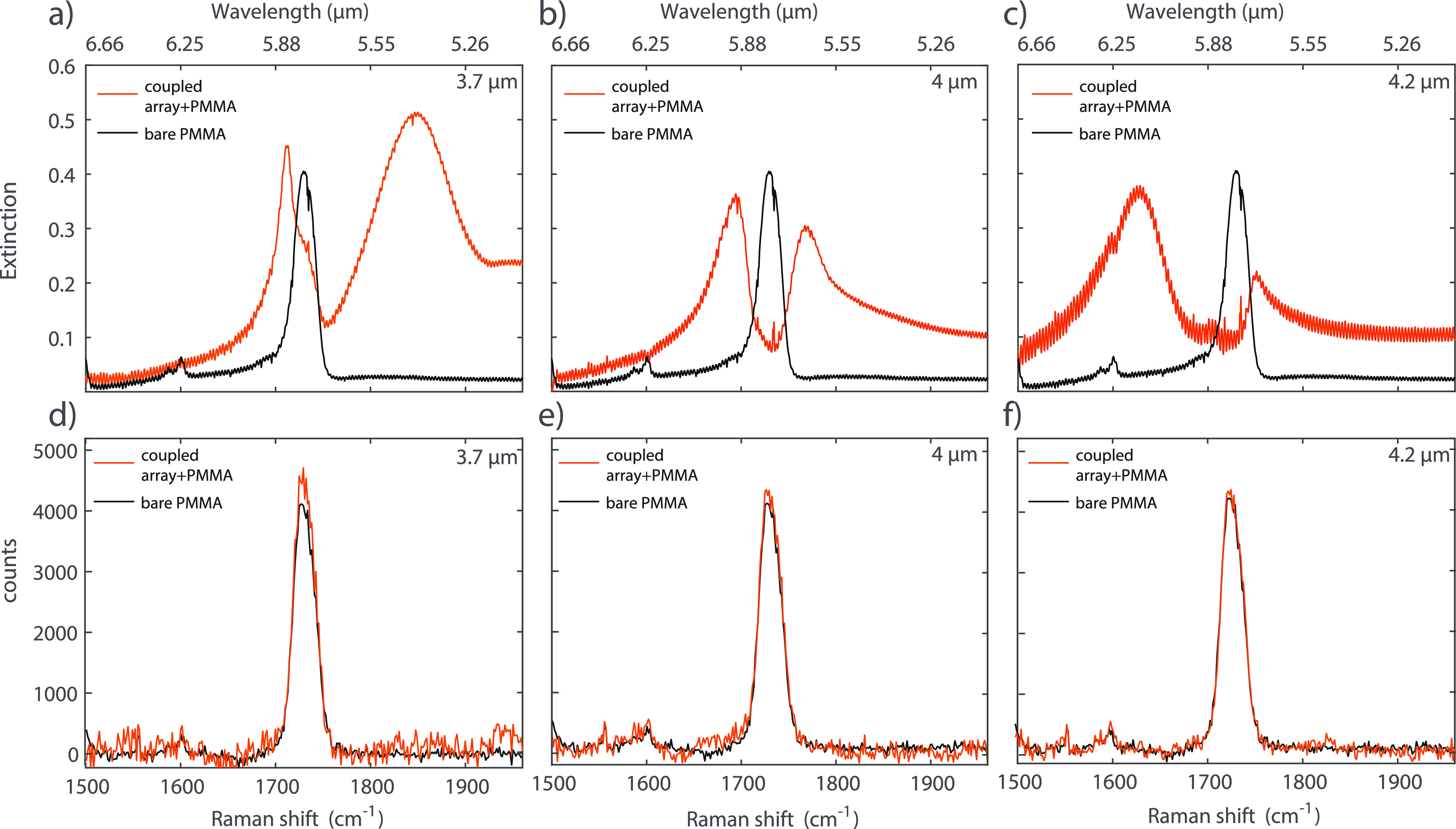
FTIR and Raman spectra of the bare PMMA layer and the coupled systems with different SLR-C=O detunings. FTIR (a) and Raman (d) spectra for the array with a period of 3.7 μm; the black spectra are reference measurements on the bare PMMA layer, while the orange spectra are the measurements on the coupled array with PMMA. FTIR (b) and Raman (e) spectra for the array with a period of 4 μm. FTIR (c) and Raman (f) spectra for the array with a period of 4.2 μm.

FTIR and Raman spectra for selected arrays are shown in Figure 5. The Raman spectra are measured by illuminating with a 514 nm laser with a spot size of around 5 μm corresponding to an area on the array larger than the unit cell of the metasurface. These measurements are performed with an integration time of 5 min, and they are repeated three times for each array to improve the signal-to-noise ratio. Figure 5a–c shows the measured IR extinction spectra of the arrays with periods of 3.7, 4, and 4.2 μm, respectively. The spectra of the arrays coupled with the PMMA layer (orange curves) show the two polaritons. The extinction of a bare PMMA layer with the same thickness as in the coupled system is also shown for comparison (black curves). The arrays with periods 3.7 and 4.2 μm have their SLR blueshifted and redshifted, respectively, from the C=O energy. The detuning of the SLRs affects the coupling strength and the polariton lifetime, as illustrated by the broadening of the upper and lower polaritons for the two arrays. The 4 μm period array presents an almost perfect overlap between its SLR and the PMMA carbonyl peak. While the VSC is clearly visible in the IR extinction spectra for the coupled arrays, as shown in Figure 5a–c, we do not observe any indication of lower and upper polaritons in the Raman spectra for the tuned (Figure 5e) or detuned (Figure 5d,f) arrays.

An effect of VSC on the carbonyl peak of PMMA was measured by Ahn et al.^44^ They report a softening of the blue side of the C=O bond if coupled with the cavity mode of a Fabry–Perot cavity. However, the modification of the PMMA C=O bond stretching is not present in our systems, as visible in Figure 5d–f. However, the strongly coupled samples show a small peak intensity enhancement of the Raman signal with respect to the bare PMMA due to the field enhancement of the SLR (see Figure 5d–f and the Supporting Information, Figures S6 and S7). This increase in intensity is in line with reported measurements performed in microcavities and with localized plasmon resonances.^45,46^

**Figure 6:**
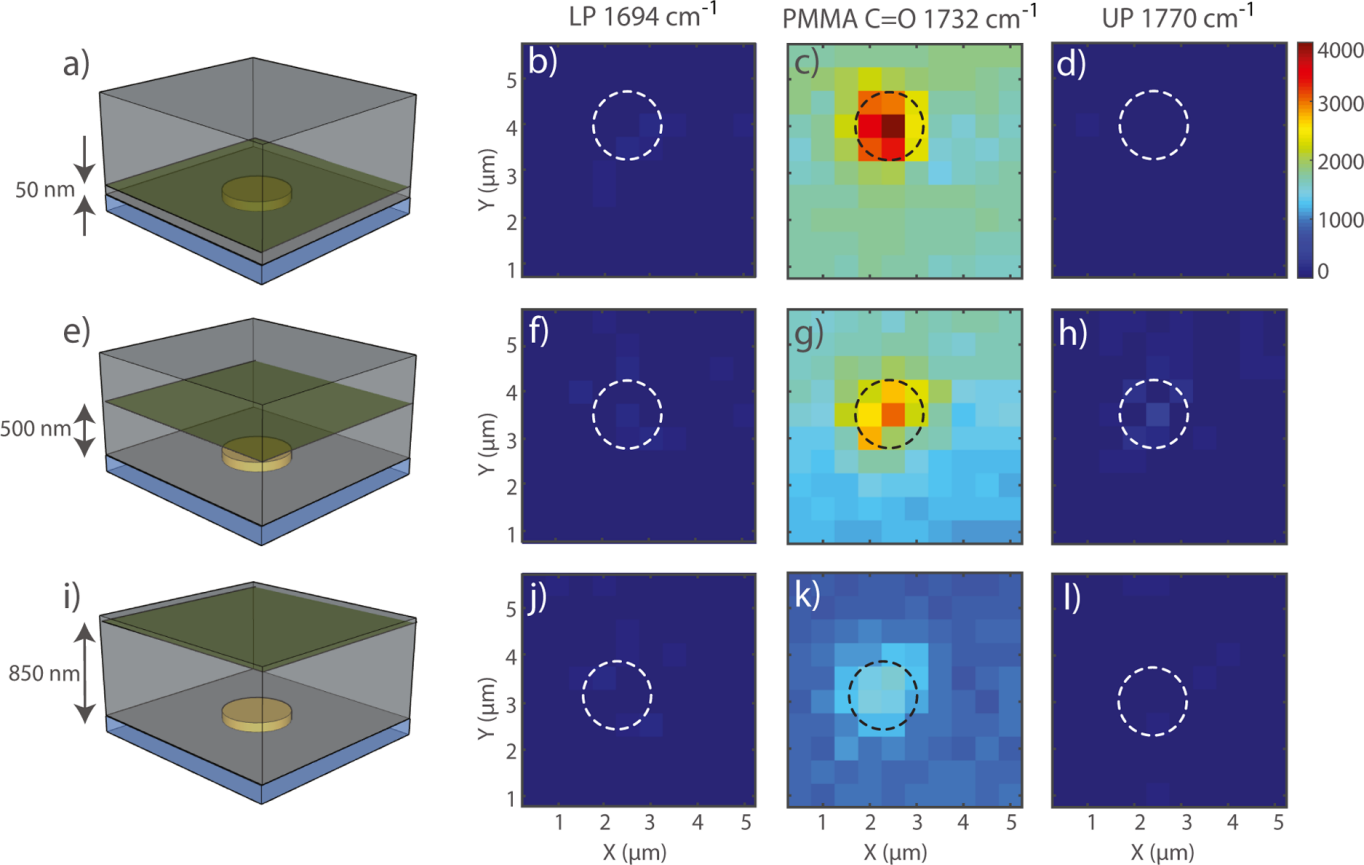
Intensity maps of the Raman signal at the wavenumber 1694 cm^–1^ (LP), 1732 cm^–1^ (PMMA C=O), and 1770 cm^–1^ (UP) in the unit cell of the array. The measurements are performed on the array with a 4 μm period. The imaging is performed at heights of 50 nm (a), 500 nm (e), and 850 nm (i). (b) Shows the maps measured at the energy of the lower polariton and a height of 50 nm. (c) Shows the maps measured at the energy of the PMMA carbonyl peak and a height of 50 nm. (d) Shows the maps measured at the energy of the upper polariton and a height of 50 nm. (f–h) show the upper, the C=O bond, and the lower polariton (respectively) energy maps measured at 500 nm. (j–l) show the upper, the C=O bond, and the lower polariton (respectively) energy maps measured at 850 nm.

The absence of polaritons in the Raman signal could be explained by a large number of dark vibronic states and uncoupled molecules in our strongly coupled arrays, which dominate the measured signal. These molecules could be at positions of low SLR field enhancements (Figure 2c,e) or with a vibration dipole moment oriented orthogonal to the field, thus interacting weakly with the SLR.^47,48^ To investigate this possibility further, we have mapped the Raman signal at the PMMA C=O bond resonance energy and at the energies of the lower and upper polariton wavelengths over a region slightly larger than a unit cell of the array—5 μm × 5 μm with 500 nm steps. The array under investigation has a period of 4 μm. We use a diffraction-limited laser beam (514 nm, ∼6.5 mW) in a confocal Raman microscope. This setup allows us to scan the beam both across the *xy* plane and at different heights in the polymer to map any local change in the Raman spectrum that could be attributed to strong coupling. Using a confocal pinhole, these measurements are performed at heights of 50, 500, and 850 nm from the CaF_2_–PMMA interface (Figure 6a,e,i, respectively). These heights are chosen based on the field profiles shown in Figure 2d,e.

The PMMA-carbonyl peak intensity is stronger on top of the particle for the three heights under study, as apparent in Figure 6c,g,k. This enhancement of the intensity of the C=O Raman signal is higher close to the particle and caused by the larger SLR field on the top of the particle, in agreement with Figure 2e. Figure 6b,f,j (for the lower polariton) and 6d,h,l (for the upper polariton) do not show any evidence of vibro-polaritons, in accordance with the measurements of Figure 5d,e,f. Some regions of high intensity are present on top of the gold disk, especially in the measurements performed at a height of 500 nm (Figure 6h), and are most likely caused by gold fluorescence (see the Supporting Information, Figure S8). The absence of polaritons in the Raman spectra and micro-Raman maps, even in regions with high SLR field enhancement, confirms previous results in Fabry–Perot cavities.^38,49^ A possible explanation for this absence could be found in the less-than-ideal spatial overlap of the electromagnetic modes at optical and infrared wavelengths.^42,43,50^ This is a consequence of the inhomogeneity of the field profile in our periodic array, as shown in Figure 2c–e. However, it has been shown that even in Fabry–Perot cavities strongly coupled to molecular vibrations, with rather homogeneous field distributions and good mode overlap, vibro-polariton signatures in Raman spectra remain elusive. While our results contribute to disentangling the coupled and uncoupled molecules’ contribution to the Raman signal by probing regions with different field enhancements, they do not provide a definitive explanation for the absence of polaritons in the measured Raman spectra.

## Conclusions

We have fabricated and characterized square arrays of gold microdisks sustaining SLRs that strongly couple to the C=O bond vibration in PMMA films. VSC was determined with IR extinction measurements and confirmed with numerical simulations. A Rabi splitting of 76 cm^–1^, larger than the loss and decoherence rates, was measured. By tuning the period of the array, we can also control the energy detuning between the SLRs and molecular vibration that defines the coupling. Measurements at normal incidence of the extinction for different arrays show the characteristic polariton anticrossing of strongly coupled systems. Raman spectra of strongly coupled samples do not show the formation of polaritons. To confirm these results and minimize the contribution of dark states or uncoupled molecules, we have mapped the Raman signal over the unit cell and at different heights using confocal Raman microscopy. These measurements contribute to clarifying the controversy about the VSC in Raman spectra in Fabry–Perot cavities and suggest that vibrational ultrastrong coupling is necessary to alter the total Raman cross-section.^42^ Future work could explore the possibility of achieving ultrastrong coupling between molecular vibrations and metasurfaces and probe this regime with Raman experiments. In fact, it has been shown that, under ultrastrong coupling, the Raman signal of the system is slightly enhanced.^43^ To achieve this regime, it is possible to use periodic arrays of microstructures with extreme field confinement, such as particle dimers.^51^

## Supporting Information Available

Calculations of the RAs of the array with a 4 μm period, microparticle ellipticity calculation, extinction measurement of IR spectra of the fabricated arrays, Raman spectra of the fabricated arrays, Raman spectra of the gold background, and absorption and scattering efficiencies of the single gold disk (PDF)
Additional figures (ZIP)
