# Supplementary material for: Chasing Vibro-Polariton Fingerprints in Infrared and Raman Spectra Using Surface Lattice Resonances on Extended Metasurfaces
Source: J Phys Chem C Nanomater Interfaces. 2022 Apr 18;126(16):7143–51. doi: 10.1021/acs.jpcc.2c00779 (PMC9059191; doi:10.1021/acs.jpcc.2c00779)
Supplement: Supplementary file 1 — jp2c00779_si_001.pdf [file jp2c00779_si_001.pdf]

Supporting Information for Chasing Vibro-Polariton Fingerprints in Infra-Red and Raman Spectra using Surface Lattice Resonances on Extended Metasurfaces

Francesco Verdelli,[†] Jeff J.P.M. Schulpen,[‡] Andrea Baldi,[¶] and Jaime Gómez Rivas[*,§]

†*Dutch Institute for Fundamental Energy Research, Eindhoven 5600HH, The Netherlands.*

‡*Department of Applied Physics, Eindhoven University of Technology, 5600MB, The Netherlands*

¶ *Vrije Universiteit Amsterdam, Amsterdam 1081HV, The Netherlands*

§*Institute for Photonic Integration, Department of Applied Physics, Eindhoven University of Technology, 5600MB, The Netherlands*

E-mail: j.gomez.rivas@tue.nl

Table of contents

- **S1. Scattering and absorption efficiencies of the gold microdisk**
- **S2. Rayleigh anomalies calculation**
- **S3. Particle ellipticity**
- **S4. FTIR measurements at different positions**

• **S5. Raman measurements**

S1. Scattering and absorption efficiencies of the gold microdisk

The scattering and absorption efficiencies are calculated using FDTD. We simulate a single gold disk with diameter of 1.4 μm and height of 50 nm.

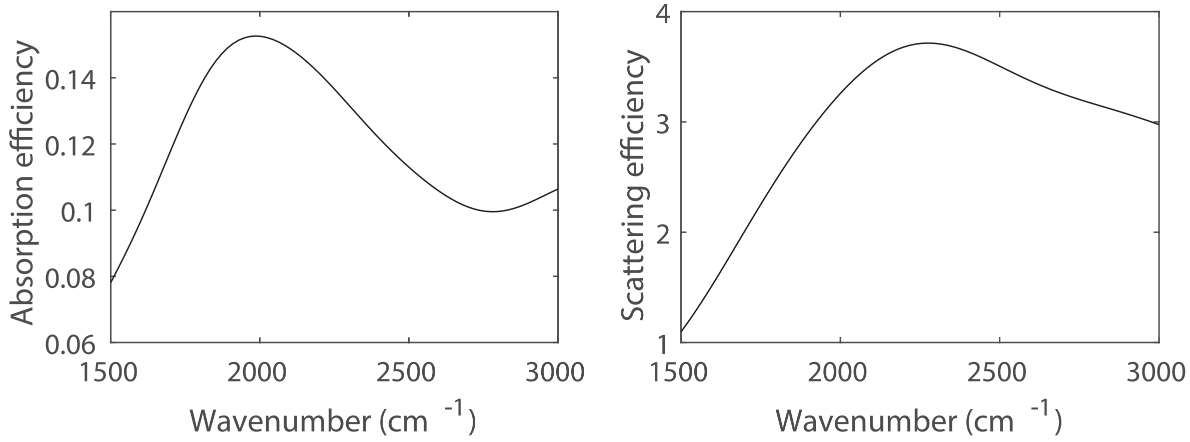

Figure S1: FDTD simulations of the a) absorption cross-section and b) scattering cross-section of the single gold disk.

S2. Rayleigh anomalies calculation

A wave illuminating a square array of particles acquires an additional momentum. This momentum is an integer of $|\mathbf{G}_x| = |\mathbf{G}_y| = 2\pi/a$, where a is the period of the array. When the wave is diffracted in the plane parallel to the array surface, a feature in the transmission spectrum of the array is observed: the Rayleigh anomaly (RA). This phenomenon occurs when

$$\mathbf{k_{out}} = \mathbf{k_0} \sin\theta + i\mathbf{G}_x + j\mathbf{G}_y, \tag{1}$$

where θ is the incident angle and (i,j) are integers defining the diffraction order. The different RAs can be calculated using the following equation for a plane wave incident along the x-axis

$$k_{\text{out}} = \sqrt{(k_0 \sin\theta)^2 + (i^2 + j^2)\left(\frac{2\pi}{a}\right)^2 + 2k_0 \sin\theta \frac{2\pi}{a} i} \tag{2}$$

The lowest order RAs calculated for a square array with a=b=4 μm and $k_0 \sin\theta$ along the x-axis are plotted in Fig. S2.

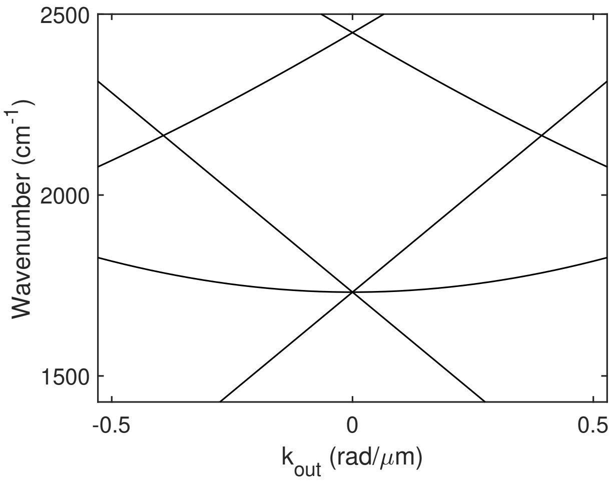

Figure S2: Rayleigh anomalies for a gold array with period of 4 μm embedded in a medium with refractive index of 1.44. The calculated orders are (0,±1),(±1,0) and (±1,1).

S3. Particles ellipticity

The particles are not perfect cylinders. An analysis with the open source software ImageJ of the AFM images (Fig. S3) reveals that the particles have an ellipsoidal shape. The average ellipticity parameter extracted from the fit is 0.795, as calculated using the equation

$$\textbf{ellipticity} = 4\pi\frac{[\text{Area}]}{[\text{Perimeter}]^2}. \tag{3}$$

If the ellipticity is 1.0, the particles are perfect cylinders. In our case the average ellipticity is roughly 0.8, which means that the particles have a long and a short axis effecting the spectral position of the surface lattice resonance of the array.

S4. FTIR measurements at different positions

The IR extinction ($1 - T/T_{ref}$) spectra of periodic gold arrays with different period (ranging from 3.7 μm to 4.2 μm) are shown. The measurements are repeated for each array in different regions to determine the error of the polariton energy. The bare PMMA spectra is used as reference, T_{ref}, to remove the contribution of uncoupled molecules to the signal.

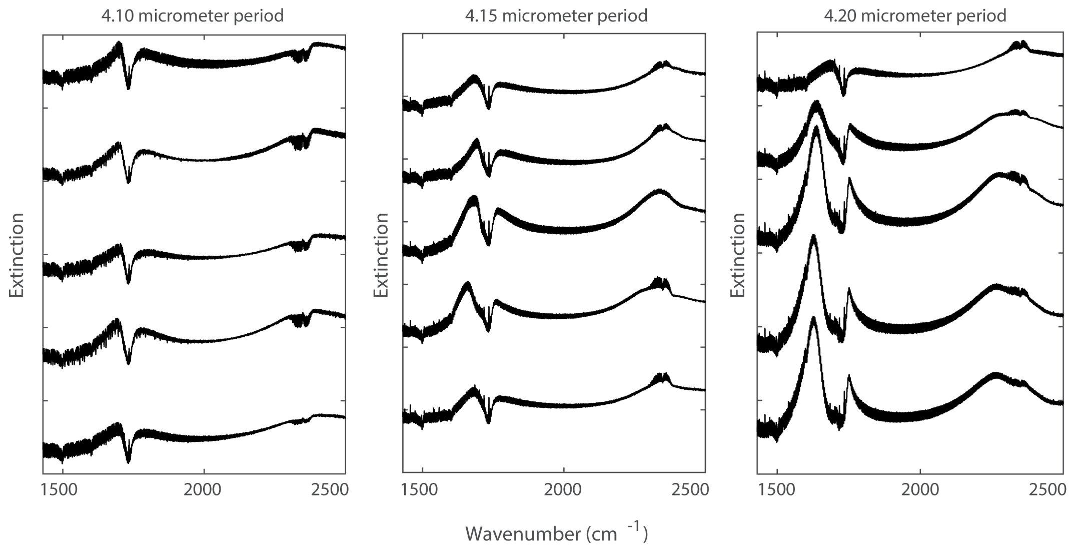

Figure S3: FTIR extinction measurement at different positions on the arrays with periods 4.2 μm, 4.15 μm and 4.1 μm. Each spectra is an average of 60 scans. Each array was measured in 5 different positions with different sample quality. The measurements were normalized using the bare PMMA extinction measured outside the array. These spectra were used to determine the error on the polariton energies. This error was added to the spectral resolution of the instrument, which is 0.5 cm^{-1}.

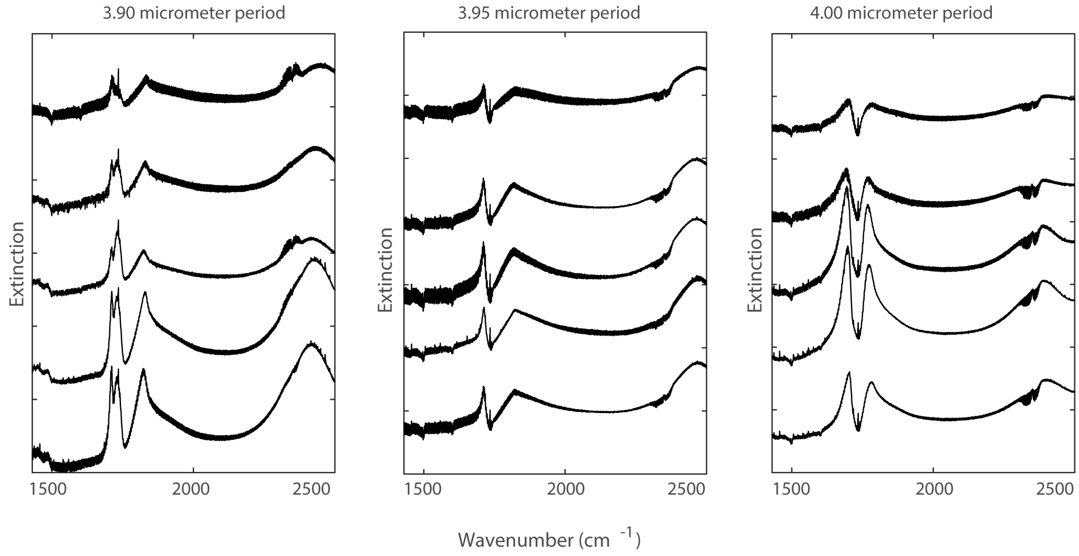

Figure S4: FTIR extinction measurement at different positions on the arrays with period of 4 μm, 3.95 μm and 3.9 μm. Each spectra is an average of 60 scans. Each array was measured in 5 different positions with different sample quality. The measurement were normalized using the bare PMMA extinction measured outside the array. These spectra were used to calculate the error on the polariton energies. This error was added to the spectral resolution of the instrument, which is 0.5 cm^{-1}.

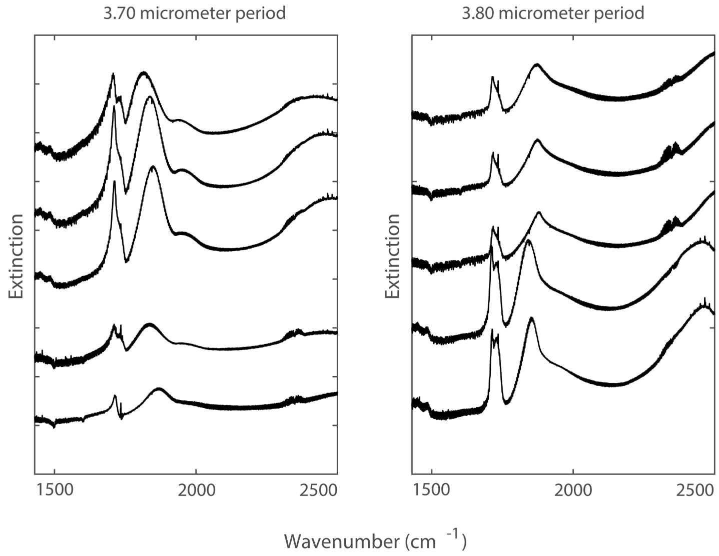

Figure S5: FTIR extinction measurement at different positions on the arrays with period of 3.8 μm and 3.7 μm. Each spectra is an average of 60 scans. Each array was measured in 5 different positions with different sample quality. The measurement were normalized using the bare PMMA extinction measured outside the array. These spectra were used to calculate the error on the polariton energies. This error was added to the spectral resolution of the instrument, which is 0.5 cm^{-1}.

S5. Raman measurements

The Raman measurement were performed using an InVia Renishaw microscope. The light source is a 514nm laser at 50% power ($\sim$ 6.5 mW). The measurements were integrated for 5 minutes and repeated several times to improve the signal to noise ratio.

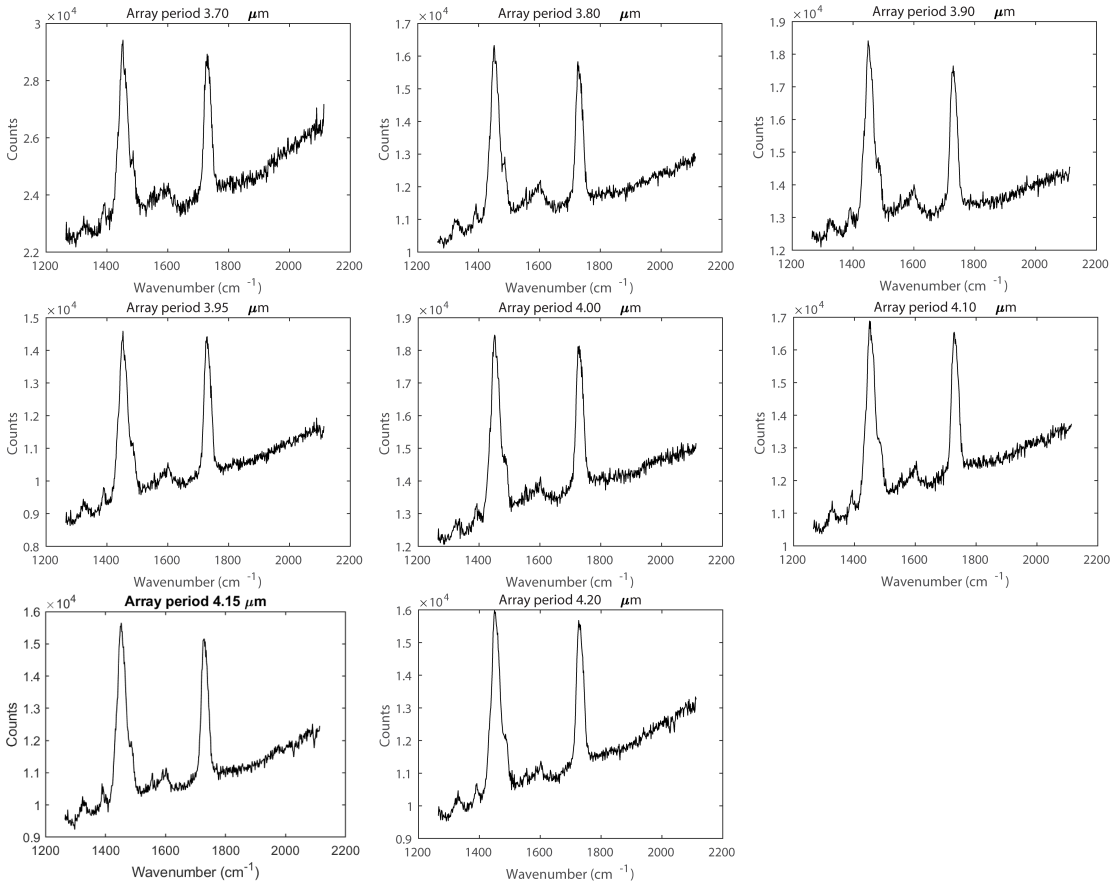

Figure S6: Raw Raman spectra of PMMA on top of metasurfaces with different pitches. The peak at 1730 cm^{-1} is associated with the carbonyl vibration of PMMA. The peak at 1444 cm^{-1} is associated with the C-H deformation.

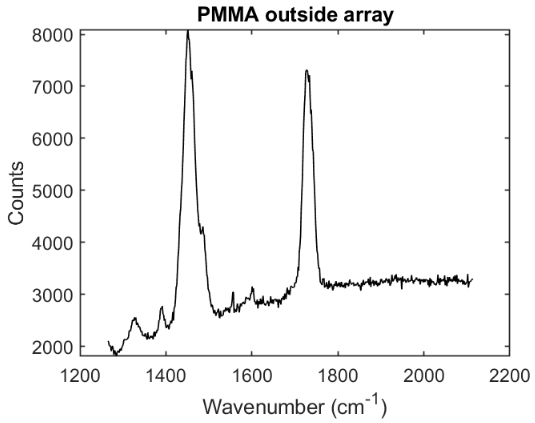

Figure S7: Raman spectrum of PMMA on top of the CaF_2 substrate.

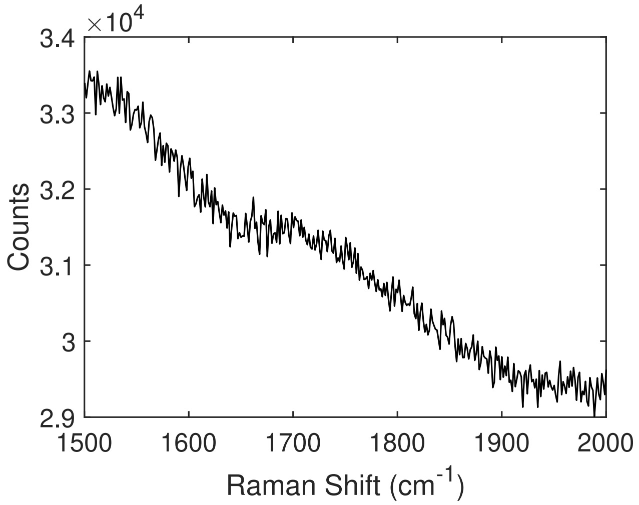

Figure S8: Measurement of the Raman background of a 50nm gold film.
