# Supplementary figures and images for: Chasing Vibro-Polariton Fingerprints in Infrared and Raman Spectra Using Surface Lattice Resonances on Extended Metasurfaces
Source: J Phys Chem C Nanomater Interfaces. 2022 Apr 18;126(16):7143–51. doi: 10.1021/acs.jpcc.2c00779 (PMC9059191; doi:10.1021/acs.jpcc.2c00779)

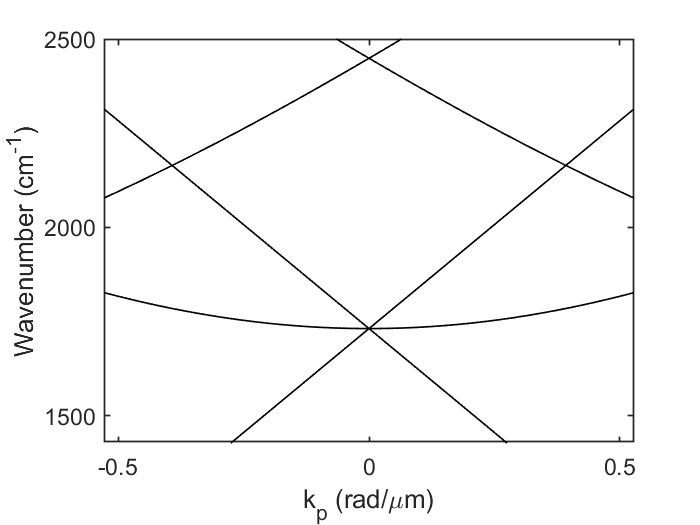

Supplement: Supplementary file 2 — jp2c00779_si_002.zip [file jp2c00779_si_002.zip › Rayleigh_anomalies.png]

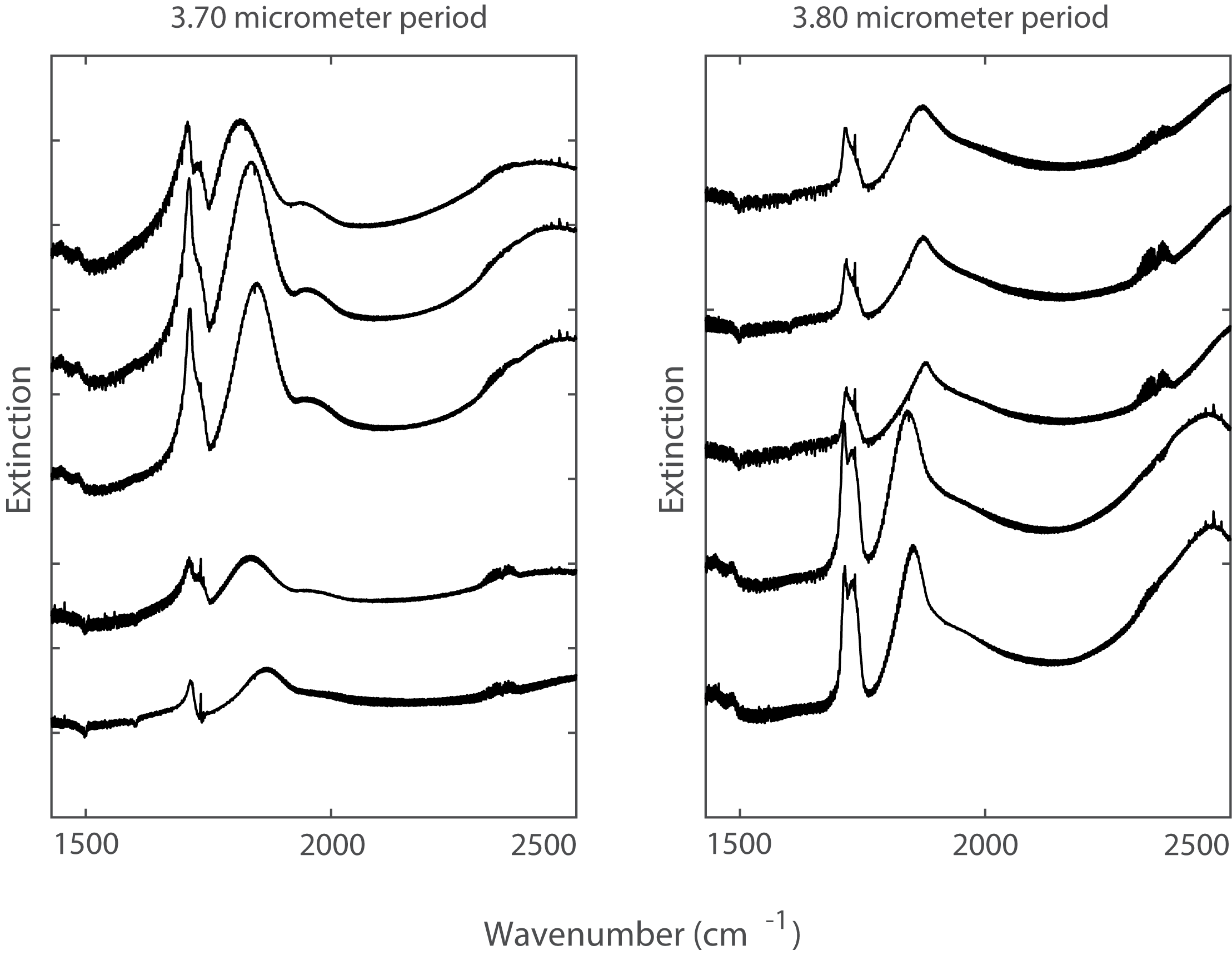

Supplement: Supplementary file 2 — jp2c00779_si_002.zip [file jp2c00779_si_002.zip › 380_370_Allspectra.png]

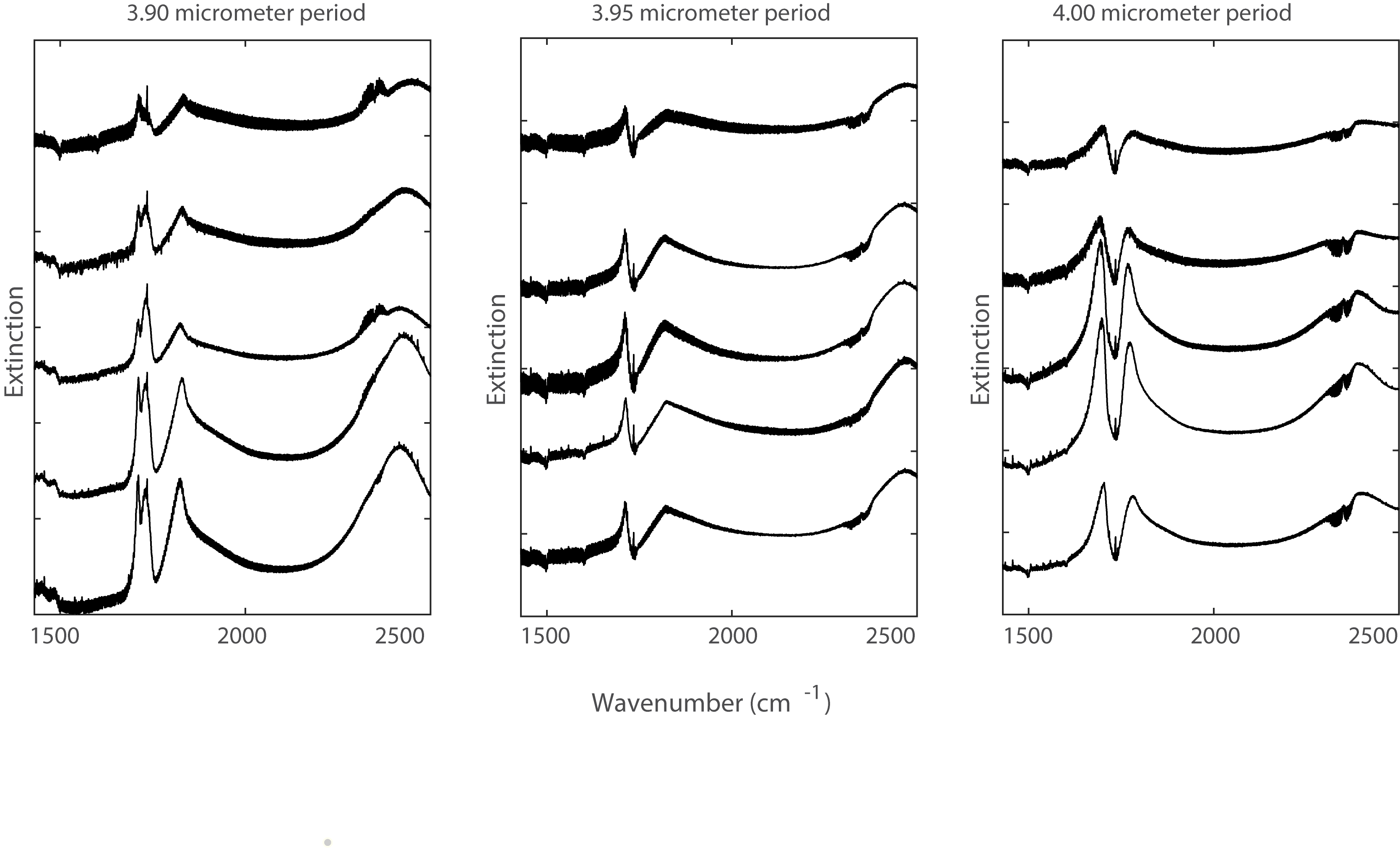

Supplement: Supplementary file 2 — jp2c00779_si_002.zip [file jp2c00779_si_002.zip › 400_395_390_Allspectra.png]

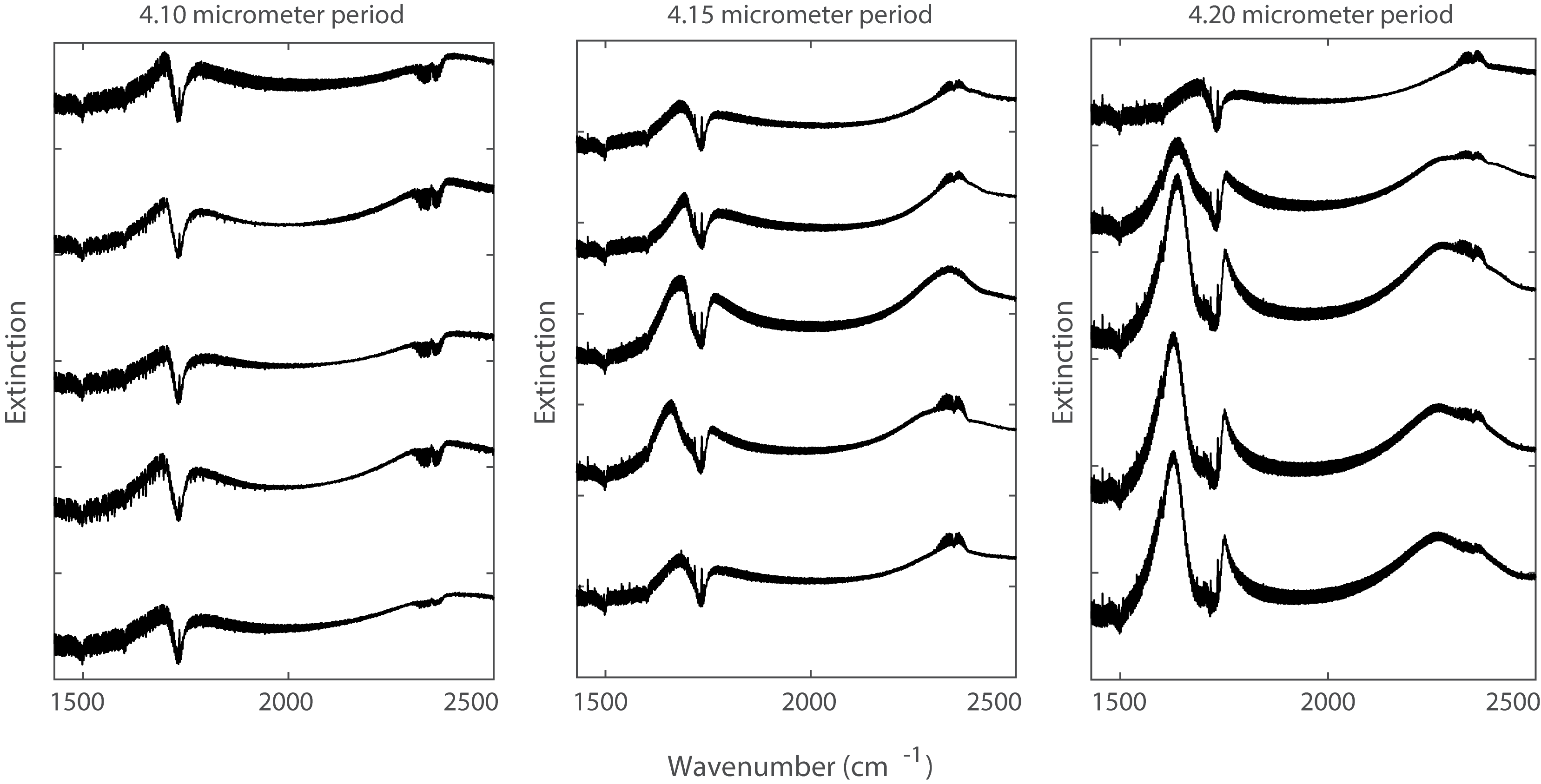

Supplement: Supplementary file 2 — jp2c00779_si_002.zip [file jp2c00779_si_002.zip › 420_415_410_Allspectra.png]

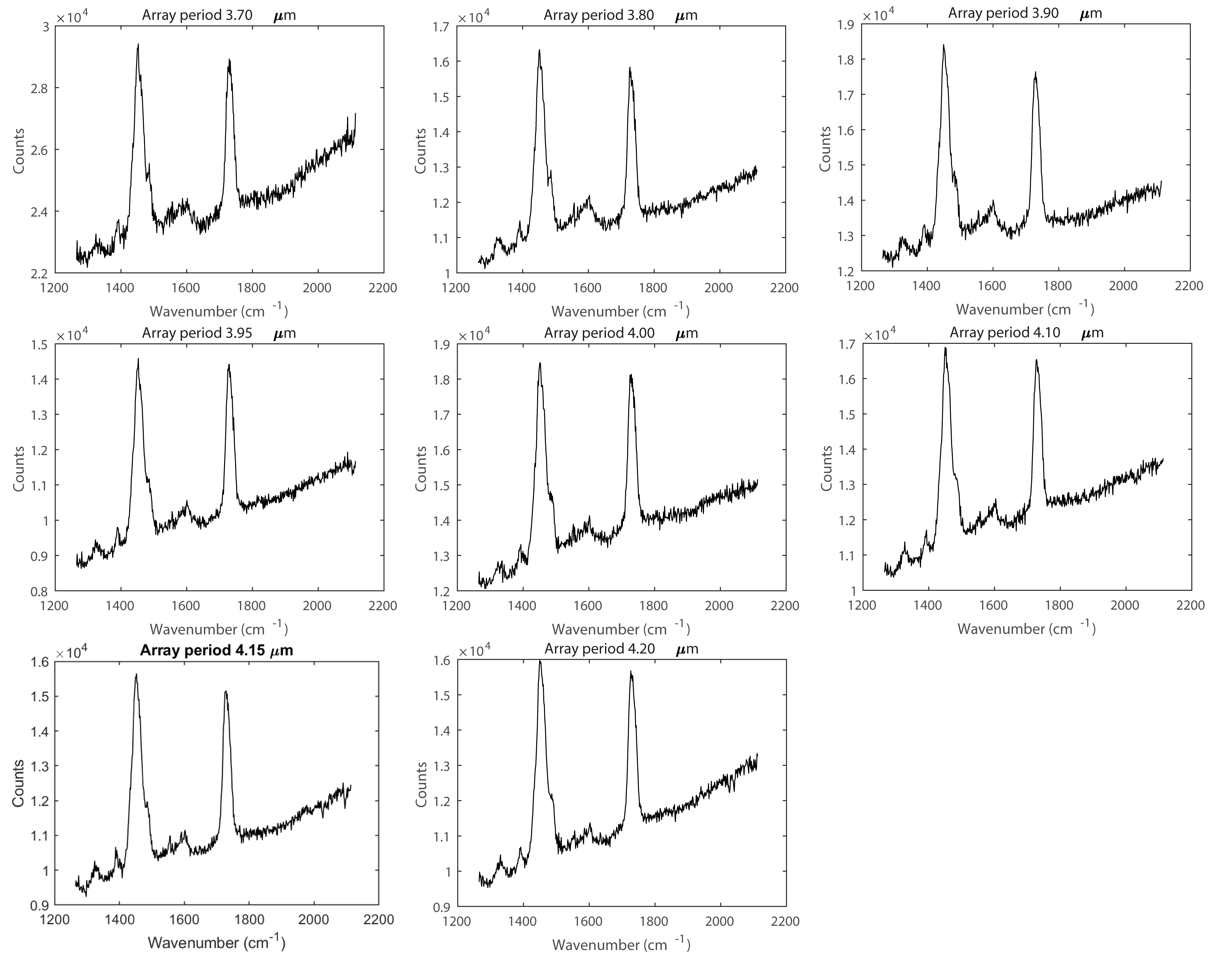

Supplement: Supplementary file 2 — jp2c00779_si_002.zip [file jp2c00779_si_002.zip › Raman4SI.png]

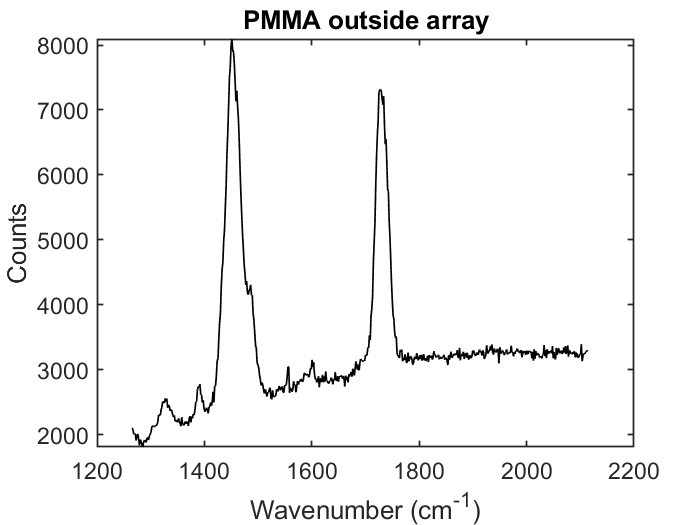

Supplement: Supplementary file 2 — jp2c00779_si_002.zip [file jp2c00779_si_002.zip › Pmma_offarray.png]

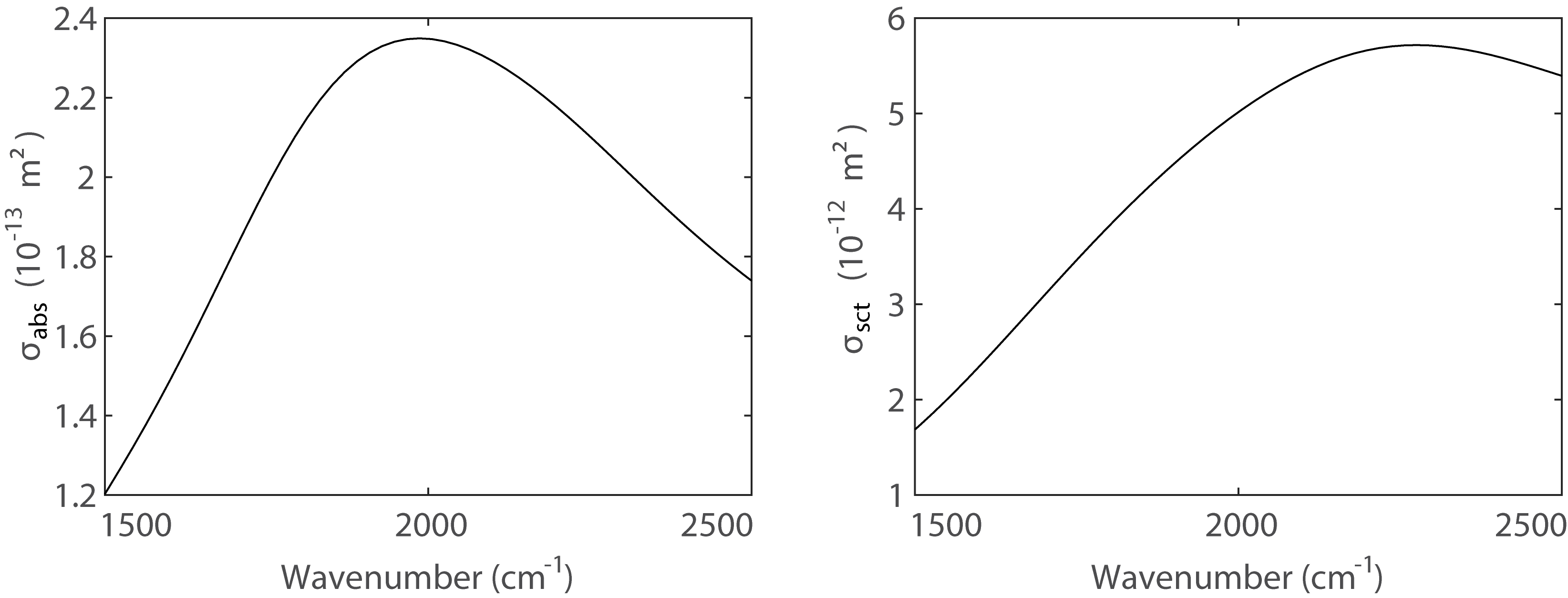

Supplement: Supplementary file 2 — jp2c00779_si_002.zip [file jp2c00779_si_002.zip › scattering_absortptioncross.png]
